# Large bilateral adrenal metastases in non-small cell lung cancer

**DOI:** 10.1186/1477-7819-2-37

**Published:** 2004-11-13

**Authors:** Charisios Karanikiotis, Apostolos Anto Tentes, Sotirios Markakidis, Konstantinos Vafiadis

**Affiliations:** 1 Department of Medical Oncology, 424 Army General Hospital, Thessaloniki, Greece; 2 Surgical Department, Didimotichon General Hospital, Didimotichon, Greece; 3 Department of Radiology, Didimotichon General Hospital, Didimotichon, Greece

## Abstract

**Background:**

The adrenal gland is one of the common sites of metastasis from primary lung cancer. Adrenal metastases are usually unilateral however bilateral adrenal metastases are seen in 10% of all lung cancer patients; of these 2–3% occurs at the initial presentation of non-small cell lung cancer. Secondary tumors can disrupt the structure and function of the adrenal. This can lead to adrenal hemorrhage, which constitutes a life threatening hazard for the patient.

**Case presentation:**

A 59-year-old male presented with persisting abdominal pain. His initial work-up revealed significant anemia, an invasive process in the right upper lobe of the lung and large masses of heterogeneous texture, with hemorrhagic and necrotic elements in both adrenal glands. A biopsy confirmed it to be a large-cell carcinoma of the lungs. The patient developed severe leukocytosis akin to the paraneoplastic syndrome and died suddenly five days after the administration of chemotherapy.

**Conclusion:**

Intratumoral hemorrhage is a rare but life threatening complication of adrenal metastases and should be treated as soon as it has been diagnosed. If adrenalectomy is not feasible, combination chemotherapy should be applied as in metastatic disease. For choosing the appropriate chemotherapeutic regimen it is important to accurately achieve the diagnosis.

## Background

The adrenal gland is a common site for metastases in breast, lung and renal cell carcinomas, melanoma, and lymphoma [1]. Adrenal metastasis, at the initial diagnosis of non-small cell lung cancer, occurs in less than 10% of lung cancer patients [2]. Most cases involve solitary, unilateral, small asymptomatic lesions, incidentally discovered by CT-scan of the upper abdomen during a staging evaluation. Bilateral adrenal metastases are observed in less than 3% of patients with lung cancer. Again, most cases involve small, asymptomatic lesions. Intratumoral hemorrhage is a rare but serious complication of adrenal metastases. We report a case of non-small cell lung cancer with large bilateral adrenal metastases complicated with intratumoral hemorrhage and paraneoplastic leukemoid reaction.

## Case presentation

A 59-year-old male, working in a quarry, was admitted to the hospital complaining of abdominal pain persisting for 45 days, afternoon fever, fatigue and 8 kg weight loss during the last month. Previous medical history was non contributory. He was a heavy smoker for 45 years.

At first assessment, the patient presented with disseminated dry rales and decrease of vesicular murmur in the right upper lung field. Following abdominal examination, palpable smooth surface masses were discovered in both renal areas. Laboratory tests demonstrated a significant anemia with normocytic features (hematocrit 24%, Hb 7.2 mg/dl).

**Figure 1:**
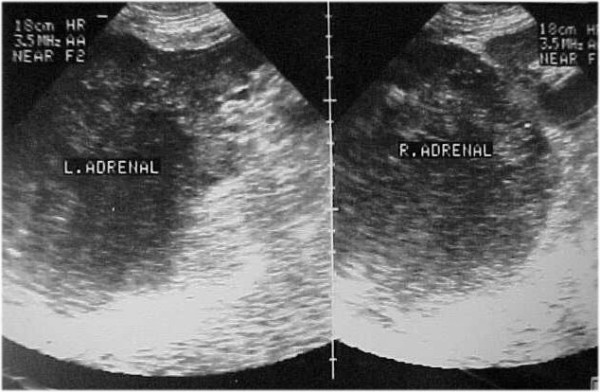
Abdomen ultrasound showing large masses with foci of hemorrhagic necrosis in both adrenal glands.

Chest X-ray revealed a consolidated area in the right upper lobe of the lung. Abdomen ultrasound demonstrated large masses with foci of hemorrhagic necrosis in both adrenal glands (Figure 1).

**Figure 2:**
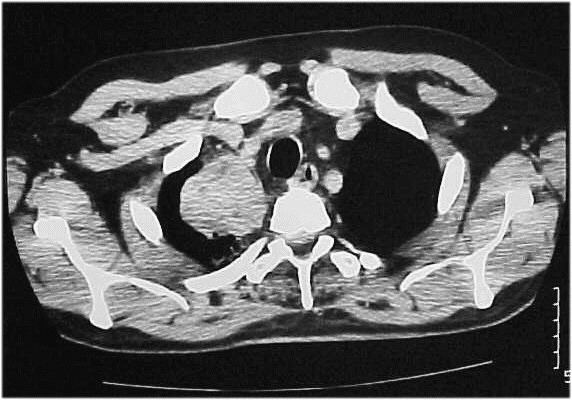
CT-scan of the chest showing a 60 × 53 mm invasive process in the right upper lobe of the lung.

**Figure 3:**
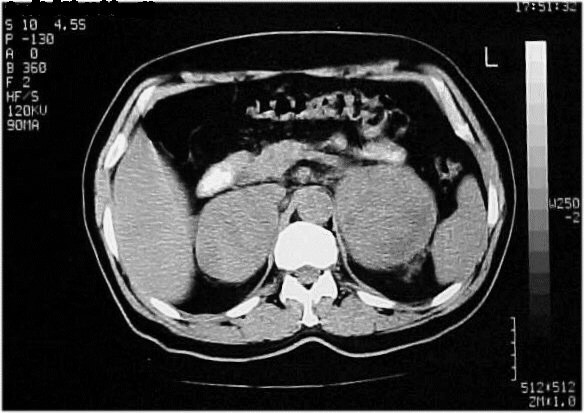
CT-scan of the abdomen showing large masses (98 × 85 mm right and 90 × 65 mm left) of heterogeneous texture, with hemorrhagic and necrotic elements within both adrenal glands.

Computed tomographic (CT) -scan revealed an invasive process in the right upper lobe of the lung (Figure 2) and large masses of heterogeneous texture, with hemorrhagic and necrotic elements within both adrenal glands (Figure 3).

**Figure 4:**
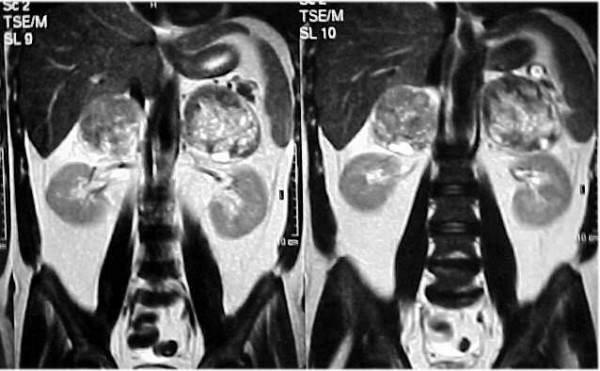
T_2 _weighted MR image showing disseminated foci of high heterogeneous signal intensity.

**Figure 5:**
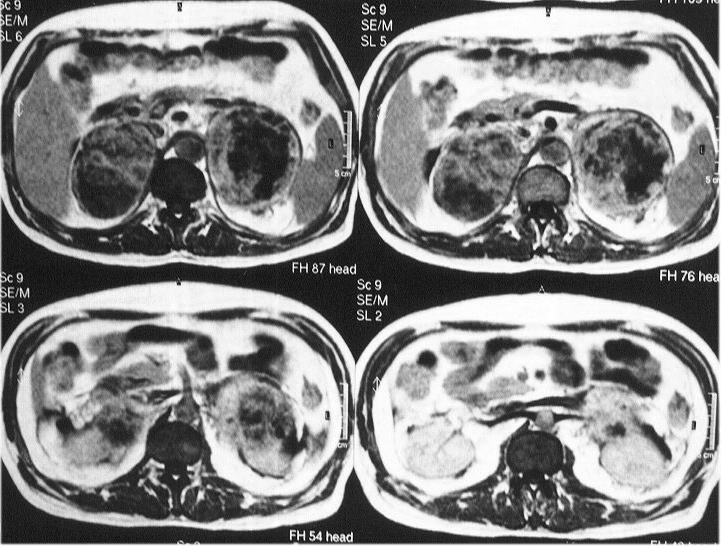
T_1 _weighted MR image showing enhancement after i.v. administration of contrast agent.

Magnetic resonance imaging (MRI) was performed which showed the masses with disseminated foci of high, heterogeneous signal intensity and pathologic enhancement after intravenous (i.v.) administration of contrast agent in both T_1 _and T_2 _weighted sequences. Radiological features were suggestive of adrenal metastasis from lung primary (Figures 4, 5). Histological examination of the biopsy specimen confirmed the diagnosis of a large-cell carcinoma of the lung. The patient showed significant deterioration and was hospitalized again for altered level of consciousness, blurring of vision, tinnitus, lability and intense fatigue. Laboratory examination demonstrated severe leukocytosis with 98,000 polymorphonuclear leukocytes, compatible with the leukemoid reaction within the framework of a paraneoplastic syndrome. Hematocrit (Ht) levels dropped significantly a second time (Ht: 21%, Hb: 6.9 mg/dl), the biochemical parameters were normal, while the adrenocorticotropic hormone (ACTH) stimulation test excluded the possibility of cortical adrenal failure (serum cortisol basal value: 9μg/dl and 34μg/dl six hours following intramuscular (i.m.) administration of 1 mg of ACTH). A repeat abdominal ultrasound revealed numerous hemorrhagic elements inside the adrenal masses. The patient received urgent chemotherapy with paclitaxel and carboplatin, with simultaneous supportive treatment. Following chemotherapy, leukocytes decreased progressively, while symptoms related to the central nervous system (CNS) improved, however, the patient died suddenly on the fifth day following chemotherapy. Autopsy was not performed. The physician's on-call medical report documented a typical cardiac arrest, without evidence of acute hemorrhage in adrenal mass.

## Discussion

Adrenal metastases in the natural history of a malignant neoplasm occur in 20 – 45% of cancer patients, depending on the localization of the primary site [3]. Up to 40% of patients with non-small cell lung cancer develop unilateral or bilateral adrenal metastases, as the carcinoma progresses [4].

The differential diagnosis of an adrenal mass incidentally discovered during imaging examination of the abdomen in patients with non-small cell lung cancer, usually comprises one or more of the characteristics detailed in Table 1.

**Table 1:** Differential diagnosis of incidentally discovered adrenal mass on Imaging

• Nonfunctional adenoma
• Adrenal metastasis
• Primary carcinoma in adrenal glands
• Adrenal cyst
• Nonfunctional pheochromocytoma
• Other causes (myelolipoma, lymphoma, aldosteronoma, neuroblastoma, pheochromocytoma)

More than 90% of the cases comprising this involve a nonfunctional adenoma or an adrenal metastasis.

Nonfunctional adenomas can be distinguished from adrenal metastasis with precision using modern imaging techniques, especially with MR imaging, where adenoma presents a low intensity signals in T2 sequences compared to hepatic parenchyma. In contrast, the adrenal metastasis presents higher intensity T2 sequence signals than that of the adenoma, relative to hepatic parenchyma. In CT-scan adenoma presents low intensity signals, below 10 Hounsfield units, compared to higher intensity signals in adrenal metastasis. Porte *et al*, observed that CT-scan technology has low specificity and sensitivity, which culminates in false positive and negative readings, given that 21% of true adenomas can be mistaken for metastases (density > 10 Hounsfield units) and 11% of true metastases may be mistaken for adenomas (density < 10 Hounsfield units) [5]. CT-scan has 100% sensitivity in detecting the metastatic nature of adrenal masses; however, 50% of adenomas may be mistaken as metastasis. Diagnostic accuracy of adrenal metastasis typically increased with detection of a primary tumor, as well as changes in the size and architecture of the parenchyma over time or during treatment. In equivocal cases, CT-scan-guided percutaneous biopsy of the adrenal mass is a safe and reliable method for the diagnosis of the lesion (sensitivity 81%, specificity 99%, potential complications 2.8%) [6].

In presence of adrenal metastasis the disease is staged as stage IV, which demonstrates the aggressive biological behavior and systemic dissemination of secondary lesions. These are treated as any other metastatic neoplasm and the appropriate chemotherapy is started. In select patients, where the primary lung site is surgically resectable (T1, T2 and maybe T3), with no involvement or the involvement of only the peribronchial and portal lymph nodes (N0, N1) and where the adrenal metastasis constitutes the unique indication of the disease, simultaneous adrenalectomy along with resection of the primary can increase overall survival [7]. Technically, this can be achieved with thoracoabdominal or transabdominal approaches, or laparoscopically in cases of small tumors (< 5 cm), [8]. A recent meta-analysis suggests that the latter technique, when it is feasible, is associated with fewer complications than open adrenalectomy [9]. However, adrenal metastases are usually larger than 5 cm, as in our case, which makes this approach inappropriate for general application. There is also a perceived increased risk of local tumor recurrence and intraperitoneal tumor dissemination occurring after laparoscopic resection of malignant adrenal tumors [10].

The most important complication of adrenal metastases is intratumoral hemorrhage and only nine cases have been reported previously [11]. When hemorrhage risk is high, as in the case of large metastasis, timely adrenalectomy is a necessary intervention. For resecting larger adrenal tumors in presence of bilateral involvement the transabdominal approach with extended subcostal incision (Kocher) is preferred. When this is considered technically impossible, radiation therapy can be an alternative palliative approach. During the course of the disease, our patient developed a leukemoid reaction and the performance status dropped remarkably, making it difficult to proceed with the planned surgical resection. Thus, the paclitaxel and carboplatin chemotherapeutic regimen was administered in an attempt to decrease the leucocytes count and improve the CNS symptoms. Even though the infusion of paclitaxel has been associated with cardiovascular events, there is no clear relation between the drug administration and the sudden death of our patient.

A less common complication of bilateral adrenal metastases is primary adrenal insufficiency, caused by the destruction of adrenal gland architecture and function due to the development of the tumor. The main symptoms resulting from the decreased production of steroid hormones often overlap with the general indicators caused by the primary site, hindering the proper treatment of these patients. Primary adrenal insufficiency is diagnosed when serum cortisol basal values are low (<5μg/dl), and particularly when the values fail to increase within six hours following the i.m. administration of 1 mg of tetracosactride, the synthetic analog of ACTH. The treatment comprises daily administration of glucocorticoid and mineralocorticoid for life.

## Conclusion

Bilateral adrenal metastases can be found in a small number of patients with non-small cell lung cancer. Early identification of these lesions is necessary, since adrenalectomy may increase survival in select patients. Intratumoral hemorrhage is a rare but life threatening complication and should be treated as soon as it has been diagnosed. If adrenalectomy is not feasible, combination chemotherapy should be applied.

## Competing interests

The authors declare that they have no competing interests.

## Authors' contributions

CK – conceived the study, did the literature search and coordinated the preparation of the manuscript for submission

AT – helped in literature search, reviewed and edited the manuscript for final submission

SM and KV – helped in literature search and drafted the manuscript for submission

All authors read and approved the manuscript.
